# Catastrophic climate change and the collapse of human societies

**DOI:** 10.1093/nsr/nwad082

**Published:** 2023-03-23

**Authors:** Josep Peñuelas, Sandra Nogué

**Affiliations:** CREAF, Cerdanyola del Vallès, Spain; CSIC, Global Ecology Unit CREAF-CSIC-UAB, Spain; CREAF, Cerdanyola del Vallès, Spain; CSIC, Global Ecology Unit CREAF-CSIC-UAB, Spain; Universitat Autònoma de Barcelona, Spain

The scientific community has focused the agenda of studies of climate change on lower-end warming and simple risk analyses, because more realistic complex assessments of risk are more difficult, the benchmark of the international targets is the Paris Agreement goal of limiting warming to <2°C, and the culture of climate science is to try to avoid alarmism [1]. Current fires, prolonged droughts, floods and heat waves, together with the consequent food insecurity, civil unrest and migrations, however, are opening the eyes not only of most scientists but also of most people all over the world to the need for considering, at least, the potential catastrophic effects of the collapse of ecosystems and society due to the current emergency of climate change.

The projections for the climate of the coming decades are, as we all know, worrying. The worst-case scenarios in the 2022 Intergovernmental Panel on Climate Change (IPCC) report project temperatures by the next century that last occurred in the Early Eocene, reversing 50 million years of cooler climates within two centuries. The Pliocene and Eocene provide the best analogues for near-future climates [2]. Climates like those of the Pliocene are likely to prevail as soon as 2030 and unmitigated scenarios of emissions of greenhouse gases (GHGs) will produce climates like those of the Eocene for the coming decades. This situation is particularly alarming because human societies are locally adapted to a specific climatic niche with a mean annual temperature of ∼13°C [3]. We can thus logically expect that current and future warming may easily overwhelm societal adaptive capacity.

These climate projections could be even more detrimental if models would not neglect, as they currently do, feedback in the carbon cycle and potential tipping points that could generate higher GHG concentrations [4]. Examples include the apparent slowing of dampening feedbacks such as the natural carbon-sink capacity [5,6], the loss of carbon due to increasing frequencies and intensities of fire at northern latitudes [7], droughts and fires in the Amazon [8] or the thawing of Arctic permafrost that releases methane and CO_2_ [9]. This feedback is also likely not proportional to warming, as is sometimes assumed. Instead, abrupt and/or irreversible changes may be triggered at a temperature threshold [7]. Particularly worrying is a ‘tipping cascade’ in which multiple tipping elements interact in such a way that tipping one threshold increases the likelihood of tipping another [4,10].

Climate change also interacts with other anthropogenic stressors such as changes in land use, loss of biodiversity, nutrient imbalances, pollution and an overuse of available resources that are crossing the planetary safety boundary limits and operating as a possible catastrophic mix. This mix may exacerbate society vulnerabilities and cause multiple indirect stresses such as economic damage, loss of land and water, and food insecurity that can merge into system-wide synchronous failures. These cascading effects are not only biophysical or biogeochemical, but they also affect human society, generating conflicts, political instability, systemic financial risks, the spread of infectious diseases and the risk of spillover. For example, there is evidence that the 2007−10 drought contributed to the conflict in Syria [11].

**Figure 1.**
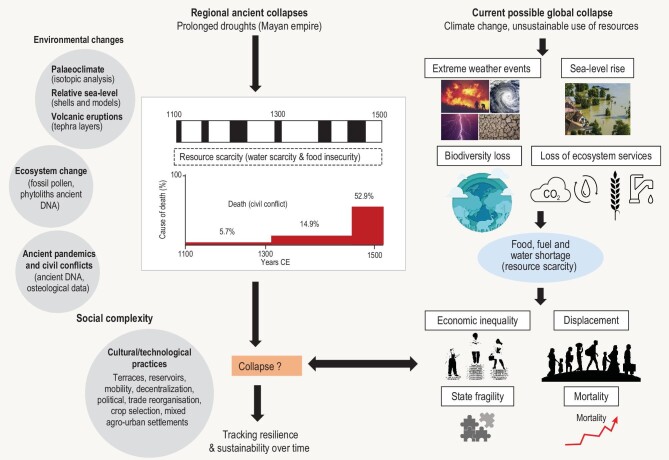
Integration of elements indicating: (i) examples of approaches to determine ancient environmental change and societal complexity; (ii) a regional/local ancient collapse described by an example from Mayapan, the largest Postclassic Mayan city [20]; and (iii) the modern potential global collapses described by Kemp *et al.* [1]. The figure shows the interconnected nature of climate change, unsustainable use of resources and civil unrest. It also highlights the relevance of ancient technologies and management strategies to mitigate the consequences of the complexity of climate change (e.g. Refs [20–25]).

Anthropogenic climate change interacting with these other stressors could thus cause a global catastrophe, in a worldwide societal collapse. Kemp *et al.* [1] have reminded us that although we have reasons to suspect it, such potential collapsing futures are rarely studied and poorly understood. The closest research is the search for evidence of tipping dynamics and estimating thresholds, timescales and impacts of potential tipping points [4]. We advocate for considering them while using the available knowledge acquired from historical and prehistorical examples of local and regional collapses, transformations and resilience of human societies also driven by climate and unsustainable use of resources (Fig. 1).

Climate change together with turbulent political rivalries and resource mismanagement that have hindered solving underlying problems have played key roles in the collapse or transformation of numerous previous human societies. Variables associated with temperature have been identified as the most strongly limiting factors to the growth of populations of European hunter-gatherers during the late Pleistocene [12]. Droughts have also been identified as drivers of stress and collapse. Thanks to analysis of tree rings from Juniperus trees coupled with ^13^C stable isotope analysis, it was possible to determine the impact of a multi-year drought on the Ancient Anatolian empire of the Hittites that collapsed >3000 years ago [13]. More recently, the Central American Mayan civilization built cities supporting populations of ≤100 000 and reached its peak about 800–1000 CE before collapsing due to a major transformation of its political and social structures and the unsustainable use of their environment, especially by deforestation and erosion [14]. Similar collapsing processes occurred in, e.g. the Roman Empire, Greenland-Iceland Norse culture [15] and Easter Island in the Pacific Ocean [16,17]

These prehistorical and historical data demonstrate that societal collapse rarely has a single cause but is often due to many biophysical and socio-economic stressors, including climate, overexploitation of resources and societal conflicts. The study of collapses of previous human societies, in many cases associated with changes in climate and resource scarcity such as those that currently characterize anthropogenic global change on our planet, sheds light on worst-case scenarios for the coming years and decades of climate change, but also on how to overcome them, on possible resistance and resilience [18,19]. For example, bioarcheological data from Mayapan (Yucatán, Mexico) indicate that Mayan political and economic structures in the shape of a network of small Mayan states persisted until the Spanish colonization, indicating some amount of regional resistance and resilience [20] (Fig. 1). In addition, the diet of the lowland Maya may have been more varied than what was previously thought. According to ethnographic and ethnobotanical assessments, the diet was potentially complemented with drought-resistant indigenous plants [21]. Other examples of cultural and technological practices are the reservoirs built around the temples of Angkor Wat, the Sri Lankan reservoirs that have provided irrigation water for cultivation for 2000 years and the terraces built to mitigate the risk of flood and to support agriculture [22,23].

The data on local and regional collapses thus provide very useful information not only on the reconstruction of past climates and past societal vulnerabilities, but also on the ways and the ancient strategies and technologies that mitigated aspects of past changes in climate, such as impacts of extreme droughts on food security and breadbaskets [24]. Analysing the mechanisms for the extreme consequences of climate change, overexploitation of resources and mismanagement of people, but also the resilience of some of these previous human societies, is strongly warranted. This needed research must also consider the differences in technologies, social complexity and cognitive levels to those cases that occurred during prehistoric and historical periods. Such analyses could help to better convince and inform managers and politicians, and society in general, to take action to mitigate and adapt to climate change, improve resilience and better prepare and diversify possible emergency responses.
